# Polysaccharides of Atractylodes Macrocephala Koidz Alleviate LPS-Induced Bursa of Fabricius Injury in Goslings by Inhibiting EREG Expression

**DOI:** 10.3390/ani15010084

**Published:** 2025-01-02

**Authors:** Shuying Gong, Bingqi Zhang, Xiang Sun, Weijun Liang, Longsheng Hong, Xiang Zhou, Wanyan Li, Yunbo Tian, Danning Xu, Zhongping Wu, Bingxin Li

**Affiliations:** 1 College of Animal Science & Technology, Zhongkai University of Agriculture and Engineering, Guangzhou 510225, China; gsy05200@163.com (S.G.); 18373917901@163.com (X.S.); 13610279293@163.com (W.L.); 13580143348@163.com (X.Z.); lwanyan88@126.com (W.L.); tyunbo@126.com (Y.T.); xdanning@126.com (D.X.); 2 College of Animal Science & Technology, Hunan Agricultural University, Changsha 410125, China; zbq2563382127@163.com; 3 College of Veterinary Medicine, South China Agricultural University, Guangzhou 510642, China; hls_6992@163.com

**Keywords:** goslings, bursa of Fabricius, polysaccharides of Atractylodes macrocephala Koidz, lipopolysaccharide, EREG

## Abstract

The bursa of Fabricius (BF) plays crucial roles in the goslings’ immune system. During waterfowl breeding, the presence of lipopolysaccharides (LPSs) in the environment can induce inflammatory damage in geese. Polysaccharides of Atractylodes macrocephala Koidz (PAMKs), as the main active component of the Chinese medicine Atractylodes macrocephala, have significant immune-enhancing effects. Accordingly, this study intended to investigate the effect of PAMKs on LPS-induced BF injury in goslings. Two hundred 1-day-old goslings (half male and half female) were selected and randomly divided into control, PAMK, LPS, and PAMK + LPS groups. The control and LPS groups were fed the basal diet, and the PAMK and PAMK + LPS groups were fed the basal diet containing PAMKs at 400 mg/kg. The goslings in the LPS and PAMK + LPS groups were injected intraperitoneally with LPS at a concentration of 2 mg/kg on days 24, 26, and 28 of this study. The control and PAMK groups were injected with equal amounts of saline. On the 28th day, 1 h after the LPS injection, the BF and serum were collected and analyzed for organ indices, cytokines, antioxidant indicators, and histological observations. Histological examination and HE staining demonstrated that the PAMK treatment ameliorated the LPS-induced BF atrophy, structural damage, increased cellular exudation, and reticulocyte hyperplasia in the goslings. The cytokine and antioxidant marker analyses in the BF cells demonstrated that the PAMK treatment mitigated the LPS-induced increase in the interleukin-1β (IL-1β), malondialdehyde (MDA), and inducible nitric oxide synthase (iNOS) levels, as well as the decrease in the transforming growth factor-β (TGF-β) and superoxide dismutase (SOD) activities. Further transcriptome sequencing identified a total of 373 differentially expressed genes (DEGs) between the LPS and PAMK + LPS groups. The KEGG enrichment pathway analysis showed that the DEGs were significantly enriched in the Toll-like receptor, p53, MAPK, GnRH, and ErbB signaling pathways. Among them, EREG played key roles in the activation of the MAPK, GnRH, and ErbB signaling pathways. Further research showed that the addition of PAMKs significantly inhibited the LPS-induced EREG expression, increased the cell viability, promoted the cell cycle entry into the S and G2 phases, and inhibited apoptosis. Meanwhile, PAMKs can reduce the protein expression of p-JNKs and c-FOS by inhibiting EREG. In summary, this study found that PAMKs could alleviate LPS-induced BF injury in goslings by inhibiting the expression of EREG.

## Simple Summary

The bursa of Fabricius, unique to birds, is critical in the immune system. LPS in waterfowl breeding environments can damage the BF in goslings. Atractylodes macrocephala polysaccharides (PAMKs), an active component from the traditional Chinese medicine Atractylodes macrocephala, enhance immune function. This study found that PAMKs mitigated the LPS-induced BF damage in goslings, reduced the inflammatory cytokine levels, and improved the antioxidant capacity. Transcriptome sequencing identified 373 differentially expressed genes, with enrichment analysis showing EREG’s key role in activating the MAPK and ErbB signaling pathways. Cell validation confirmed PAMKs’ inhibition of EREG, reduction of LPS-induced apoptosis, promotion of cell cycle progression, and decrease in apoptotic protein expression. This study concluded that PAMKs effectively alleviated the LPS-induced BF injury, providing a reference for enhancing waterfowl immunity.

## 1. Introduction

The bursa of Fabricius (BF), also known as the supracavernous bursa, is a central immune organ unique to birds [1,2]. During early embryonic development, the BF gradually forms through the interaction of ectodermal epithelial cells and mesenchymal cells. After hatching, as the bird grows, the size of the BF increases steadily, and its internal capillary network develops, providing conditions for the maturation and migration of B lymphocytes [3,4]. Lipopolysaccharide (LPS), as an endotoxin, can severely interfere with the normal physiological functions of birds. Not only does it lead to reduced feed intake, weight loss, decreased egg production, and smaller egg size but it also damages the immune system and may potentially cause death in geese and goslings [5,6]. In the goose-farming industry, environmental factors such as temperature and the scale of farming have a significant impact on the proliferation of harmful bacteria in water, which may lead to an increase in the concentration of LPS in the blood of geese [7,8]. This can affect the function of the BF, further impacting the reproductive performance of geese and the quality of goslings, which reduces the production quality. Studies found that treatment with LPS activates the TLR4-MAPK-NF-κB/AP-1 signalling pathway, which, in turn, leads to increased apoptosis and decreased cell proliferation within the bursa of Fabricius in broiler chicks, ultimately causing bursal atrophy [9]. Nevertheless, the effects of LPS on the BF in geese remain to be elucidated.

Polysaccharides of Atractylodes macrocephala Koidz (PAMKs) is an important active component of the Chinese medicine Atractylodes macrocephala, which has been recorded in the Chinese medical classic Sheng Nong’s herba. Studies showed that PAMKs have various pharmacological effects, such as hepatoprotective, antibacterial and anti-inflammatory, antioxidant, gastrointestinal function regulation, immune system regulation, and hypoglycemic effects [10,11,12]. PAMKs regulate the immune function by promoting the development of immune organs, the proliferation and activation of immune cells, the secretion of cytokines, and the maintenance of the steady state of the immune system [13,14]. Studies demonstrated that PAMKs have the ability to alleviate liver and thymus damage triggered by LPS in geese [15]. PAMKs effectively mitigate enteritis symptoms in goslings and helps restore the balance of the intestinal microbiota by modulating immune responses, enhancing antioxidant capabilities, and promoting intestinal health [16]. However, there is currently a lack of research on the effects of PAMKs on LPS-induced injury in the BF goslings. This study aimed to investigate the impact of PAMKs on the function of the BF in goslings damaged by LPS through transcriptome sequencing analysis and cellular validation experiments, providing a solid theoretical foundation and key data support for the potential application of PAMKs as immune modulators.

## 2. Materials and Methods

### 2.1. Ethics Statement

All treatments in this study were approved by the Ethics Committee of the College of Animal Science and Technology, Zhongkai University of Agriculture and Engineering (approval number: 202280301).

### 2.2. Experimental Animals and Sample Preparation

A total of 200 one-day-old Magang goslings were initially raised, comprising an equal number of males and females, all sourced from Jinye Bird Breeding (Guangdong) Co., Ltd. Following a three-day pre-feeding, these goslings were randomly allocated into four distinct groups: control group, PAMK group, LPS group, and PAMK + LPS group. It was ensured that there were no significant disparities in the average body weight across the groups at the outset of this experiment (*p* > 0.05). This study was conducted with 50 goslings in each group, with 10 goslings per replicate, and there was a total of 5 replicates. All goslings were free to feed (including vegetables) and drink. The control and LPS groups were fed the basal diet, and the PAMK and PAMK + LPS groups were fed the basal diet containing PAMKs (purity 95%, Tianyuan, Xi’an, China) at 400 mg/kg (feed). Furthermore, goslings in the LPS and PAMK + LPS groups were given intraperitoneal injections of LPS (Sigma, St. Louis, MO, USA) at 2 mg/kg (body weight) on days 24, 26, and 28 of this study, once a day [16]. Meanwhile, the control and PAMK groups were administered equal amounts of saline. On the 28th day of this study, after a 1 h treatment, the goslings were anesthetized and subsequently euthanized for the collection of the BF and serum.

### 2.3. Detection of BF Organ Index

The collected goslings’ BF organs were rinsed with saline two to three times, and then the surface was dried with filter paper for weighing. The BF organ index was calculated specifically according to the following formula: BF organ index = BF weight (g)/body weight (kg).

### 2.4. Histomorphological Observation of BF

The collected BF tissues were fixed in 4% paraformaldehyde for 48 h. Subsequently, they were paraffin-embedded and cut into 5 μm sections, which were stained with hematoxylin–eosin (HE). Optical microscopy was performed, and images were acquired using CaseViewer (2.4.0) software to observe the BF structure at 200× and 400× magnification.

### 2.5. Detection of Immunoglobulins in Serum

The gosling serum was collected and aliquoted as needed, and then stored at −80 °C to avoid repeated freeze–thaw cycles. The aliquoted serum was taken, and the instructions provided with the IgM, IgA, and IgG kits (MSKBIO, Wuhan, China) for detection were strictly followed. A plate reader was used to measure the absorbance (OD value) of each sample well.

### 2.6. Detection of Cytokines in BF

A total of 0.1 g BF was placed in 0.9 mL normal saline and cracked by a crusher. The supernatant was taken for ELISA detection. The protein concentration in the sample tissue homogenate was determined strictly according to the instructions of the BCA Protein Concentration Assay Kit (Beyotime, Shanghai, China). Subsequently, the expression levels of IL-1β, IL-6, TGF-β, and TNF-α in the BF were detected according to the instructions of the ELISA kit (MSKBIO, Wuhan, China).

### 2.7. Detection of Antioxidant Indicators in BF

A total of 0.2 g of the BF was weighed and added to 1.8 mL of PBS. The mixture was homogenized using a tissue homogenizer to obtain a 10% tissue homogenate. Following the homogenization, the sample was centrifuged at 3000 r/min for 10 min to separate the supernatant, which was then collected for further analysis. The superoxide dismutase (SOD) activity, total antioxidant capacity (T-AOC), Malondialdehyde (MDA), inducible nitric oxide synthase (iNOS), and Glutathione peroxidase (GSH-Px) were measured in BF homogenates according to the kit development instructions (Nanjing Jiancheng Bioengineering Institute, Nanjing, China).

### 2.8. RNA Extraction and Library Construction

To further investigate the mechanism of PAMK action on LPS-induced BF injury, three samples of BF from each of the LPS and PAMK + LPS groups of goslings were taken for high-throughput sequencing. First, RNA from the BF samples was isolated and purified using the reagent Trizol (Invitrogen, Carlsbad, CA, USA) according to the instructions. Next, the purity of the obtained RNA was verified by an agarose gel electrophoresis test. Next, the integrity of the RNA was examined using the Bioanalyzer 2100 system (Agilent, Santa Clara, CA, USA). Then, the captured mRNA was fragmented using the magnesium ion interruption kit (NEB, CAT.E6150, Ipswich, MA, USA) at 94 °C for 5~7 min. Subsequently, the fragmented RNA was synthesized into cDNA by reverse transcriptase (Invitrogen SuperScript™ II Reverse Transcriptase, Cat. Carlsbad, CA, USA). The composite duplex of synthesized cDNA and RNA was converted into a DNA duplex and doped with dUTP Solution (Thermo Fisher, Waltham, MA, USA) to make up the ends of the duplex DNA as flat ends, and then the fragment size was screened and purified using oligo (dT) magnetic beads. Finally, the second strand was digested with a UDG enzyme (NEB, the m0209, Ipswich, MA, USA) and the fragment was made into a cDNA library of 300 ± 50 bp size by the PCR technique. All cDNAs were double-end sequenced using an Illumina Novaseq™ 4000 (LC Bio Technology Co., Ltd. Hangzhou, China) in PE150 sequencing mode according to standard practice.

### 2.9. Identification of DEGs and Functional Enrichment Analysis

To obtain high-quality clean reads, the reads were further filtered using the software Cutadapt (https://cutadapt.readthedocs.io/en/v1.8.2/changes.html, version: cutadapt-1.9, accessed on 20 October 2022). Subsequently, the raw data from the lower machine were QC’d using FASTQ software (https://github.com/OpenGene/fastp, accessed on 25 October 2022), which included Q20, Q30, and GC content of the clean data. The alignment of sequencing data required comparison with the genome (Homo sapiens, GRCh38) using the software HISAT2 (https://ccb.jhu.edu/software/hisat2, accessed on 27 October 2022), followed by StringTie software (https://ccb.jhu.edu/software/hisat2, accessed on 28 October 2022), to assemble the genes or transcripts. This experiment utilized FPKM quantification (FPKM = total exon_fragments [mapped reads(millions) × exon length(kB)]). The R package edgeR (https://bioconductor.org/packages/release/bioc/html/edgeR.html, accessed on 29 October 2022) was used to analyze the DEGs between samples and evaluate the DEGs with two criteria: difference multiples >2-fold or <0.5-fold with *p* < 0.05.

In this experiment, all enriched DEGs were examined for Gene Ontology (GO) function using the Goseq R package. A Kyoto Encyclopedia of Genes and Genomes (KEGG) functional enrichment analysis was performed using the KOBAS online tool (http://kobas.cbi.pku.edu.cn, accessed on 2 November 2022). Values of *p* < 0.05 in the experiments represent significant differences. Finally, we visualized the results of the enrichment analysis of the GO and KEGG according to the *p*-values. GO enrichment analysis plots and KEGG pathway plots were created using the R package ggplot2 (https://ggplot2.tidyverse.org, accessed on 5 November 2022).

### 2.10. Protein–Protein Interaction (PPI) Network

The STRING 10 database (http://string-db.org/, accessed on 8 November 2022) was used to determine the relationships of DEGs at the protein level in this study. Meanwhile, the enriched genes in the key pathway were screened and imported into this database, and the results were visualized using Cytoscape_v3.2.1. In this PPI network, each dot represents a biomolecule, the lines represent interactions between biomolecules, and the sizes of the dots were adjusted according to the number of lines connected to other biomolecules: the higher the number, the larger the dots.

### 2.11. In Vitro Culture of BF Cells in Goslings

The 28-day-old goslings were anesthetized and subsequently euthanized, after which the BF was collected and its cells were extracted. The BF tissues were cleaned using PBS (Gibco, Glendale, CA, USA), and the outer connective tissue was then removed and the tissues were shredded. The shredded BF was transferred to a 15 mL centrifuge tube and DMEM (Gibco, CA, USA) (containing 10% FBS and 1% double antibody) was added, and the tissue sediment at the bottom of the tube was retained after repeated blowing and centrifugation. Subsequently, trypsin digest (0.25% with EDTA (Thermo Fisher Scientific Waltham, MA, USA)) equal to 20 times the volume of the tissue block was added to the centrifuge tube and blown and mixed, and then an equal volume of DMEM was added to the centrifuge tube to terminate the digestion. The precipitate was resuspended by adding DMEM (containing 10% FBS and 1% double antibody) to the extracted cell precipitate. After cell activity was detected using a cell counter (Invitrogen, Carlsbad, CA, USA), the number of cells was adjusted to 5 × 10^6^ cells/mL. In this study, the isolated BF cells were treated with LPS administration at concentrations of 0.1, 1, 10, and 100 μg/mL, respectively. Next, this study chose to administer the treatment with LPS at a concentration of 0.1 μg/mL and add PAMKs for interference on top of this. The concentrations of PAMKs were set to 1, 5, 10, 15, 20, 25, and 30 μg/mL. BF cells were cultured in 6-well plates for 24 h at 39 °C in a 5% CO_2_ cell incubator. In subsequent experiments, each experimental group consisted of three biological replicates.

### 2.12. Flow Cytometry Detection of BF Cell Cycle and Apoptosis

The cultured cells were collected in a centrifuge tube and washed with PBS. Then, 70% ethanol was added to the tubes, which was vortexed and mixed, and then stored at 4 °C for overnight fixation. After this, propidium iodide (Beyotime, Shanghai, China) was added following the manufacturer’s instructions, and the cells were incubated at 37 °C for 30 min under light-proof conditions before completing the cell cycle flow assay within 24 h. Next, the YP1/PI assay working solution (Beyotime, Shanghai, China) was added according to the reagent instructions, and the cells were incubated at 37 °C for 20 min under light-proof conditions, with the apoptosis flow detection completed within 4 h.

### 2.13. Western Blot Assay

Total protein was extracted from the BF and cells of goslings using a RIPA lysis buffer (Beyotime, Shanghai, China). Then, the protein concentration of each group of samples was measured using the BCA protein concentration assay kit (Beyotime, Shanghai, China). Each sample was denatured by adding an SDS-PAGE protein loading buffer (5×), mixed well, electrophoretically separated following the instructions, and subsequently transferred to polyvinylidene fluoride (PVDF) membranes. The PVDF membranes containing the target proteins were blocked in 5% skim milk powder, followed by incubation with primary antibodies GAPDH (Affinity, Nanjing, Jiangsu, China), EREG (ABclonal, Wuhan, China), c-FOS (ABclonal, Wuhan, China), RAS (ABclonal, Wuhan, China), JNKs (Proteintech Group, Wuhan, China), and p-JNKs (Proteintech Group, Wuhan, China) overnight, and then with secondary antibodies for 1 h. Finally, the protein expression on PVDF membranes was detected using a fully automated chemiluminescence imaging system and quantified with (Image J, 1.53a) software.

### 2.14. qRT-PCR Assay

The total RNA from each sample was reverse transcribed into cDNA using the TaKaRa reverse transcription kit (Takara Bio Inc., Kusatsu, Shiga, Japan). The reaction system was 20 μL: SYBR Green Master Mix 10 μL, RNase Free dH_2_O 7 μL, F Primer 1 μL, R Primer 1 μL, and cDNA 1 μL. The reaction program was set as follows: pre-denaturation at 95 °C for 5 min for 1 cycle and denaturation at 95 °C for 30 s, annealing at 60 °C for 30 s, and extension at 72 °C for 30 s for 40 cycles. The internal reference gene was *ACTB*, and the primers used and their sequences are shown in Table S1. The relative expression level of the mRNA of the target gene was calculated according to the following formula: relative expression level of gene = 2^−ΔΔCT^.

### 2.15. Statistical Data Analysis

The data from this experiment were analyzed for significance with one-way ANOVA and a two-tailed *t*-test using SPSS 26.0 statistical software. The results were visualized using GraphPad Prism 5.0 software. Data are presented as the mean ± standard error (SEM), and *p* < 0.05 was considered to indicate the significant difference.

## 3. Results

### 3.1. PAMKs Alleviated LPS-Induced Structural Damage of BF Tissues in Goslings

**Figure 1:**
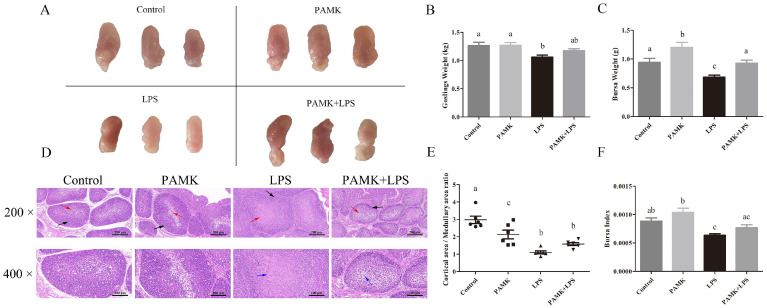
Alleviation of LPS-induced decrease in the BF organ index and histological lesions in goslings by PAMKs. (**A**) Morphology observation of the BF; (**B**) goslings’ weight; (**C**) bursa weight; (**D**) histological observation of BF (200×, 400×); (**E**) Ratio of the cortical Area to the medullary Area of the BF vesicles; (**F**) organ index of the BF. Black arrows point to the cortical area of the BF tubercle, red arrows point to the medullary area of the BF tubercle, and blue arrows point to the area of reticulocyte proliferation. Data are expressed as the mean ± standard error, and data columns labeled with different lowercase letters indicate significant differences (*p* < 0.05), and the same letter indicates that the differences are not statistically significant (*p* > 0.05).

The morphological observations show that the body size of the BF was similar in the PAMK group and smaller in the LPS group compared with the control group (Figure 1A). The results for the goslings’ weight, bursa weight, and organ index indicated that the LPS significantly reduced the BF index (*p* < 0.05), with the PAMK + LPS group showing a tendency toward upregulation (Figure 1B,C,F). The results indicate that the LPS induced a decrease in the goslings’ weight, bursa weight, and BF index, and PAMKs could alleviate these LPS-induced phenomena to some extent.

The HE staining of BF showed that in the control group, the BF vesicles were well distributed with clear definitions, and the distinction between the cortical and medullary regions was evident (Figure 1D). In contrast, the BF vesicles in the LPS group were disorganized, with narrow gaps between the vesicles, a large number of cells exuding, and obvious proliferation of reticulocytes in the medullary area. The PAMK + LPS group exhibited significant structural changes, including orderly arranged BF vesicles, increased gaps between vesicles, minimal cellular exudate, and a noticeable reduction in medullary reticulocytes. The ratio of the cortical to medullary area within the vesicles was significantly lower in the LPS group compared with the control and PAMK groups (Figure 1E).

### 3.2. Effect of PAMKs on Immunoglobulin Indices in Serum of LPS-Induced Goslings

**Figure 2:**
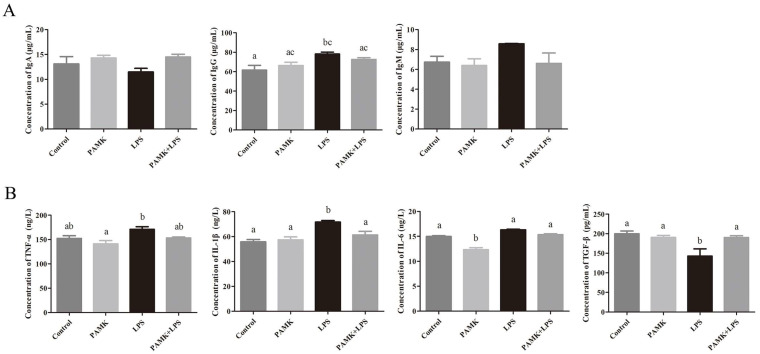
Effect of PAMKs on LPS-induced immunoglobulins and cytokines. (**A**) Serum levels of IgA, IgG, and IgM; (**B**) cytokine expression levels of TNF-α, IL-1β, IL-6, and TGF-β. Data are expressed as the mean ± standard error, and data columns labeled with different lowercase letters indicate significant differences (*p* < 0.05), and the same letter indicates that the differences are not statistically significant (*p* > 0.05).

The results of the immunoglobulin test indicate that compared with the control group, the LPS group had reduced levels of IgA and elevated levels of IgG and IgM in the serum. In contrast to the LPS group, the PAMK + LPS group exhibited higher serum levels of IgA and lower levels of IgG and IgM (*p* < 0.05) (Figure 2A).

### 3.3. Effect of PAMKs on Cytokine Expression Levels in LPS-Induced Bursa of Goslings

The cytokine test results indicate that the LPS group had elevated levels of IL-6, TNF-α, and IL-1β, and a significantly reduced level of TGF-β compared with the control group (*p* < 0.05) (Figure 2B). Additionally, the PAMK + LPS group exhibited significant decreases in the TNF-α, IL-1β, and IL-6 levels, along with a significant increase in the TGF-β levels (*p* < 0.05) (Figure 2B).

### 3.4. Effect of PAMKs on LPS-Induced Antioxidant Indexes in BF of Goslings

**Figure 3:**
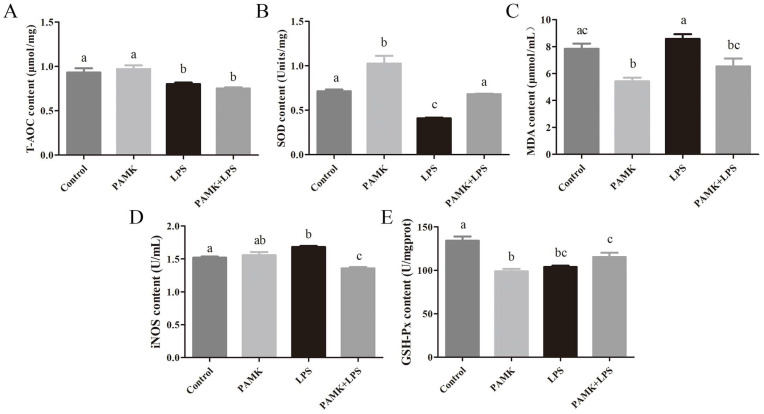
Effect of PAMKs on the LPS-induced antioxidant indexes. The levels of (**A**) T-AOC, (**B**) SOD, (**C**) MDA, (**D**) Inos, and (**E**) GSH-Px. Data are expressed as the mean ± standard error, and data columns labeled with different lowercase letters indicate significant differences (*p* < 0.05), and the same letter indicates that the differences are not statistically significant (*p* > 0.05).

Measurement of antioxidant indicators within the BF of goslings was conducted for all groups. Compared with the control group, the LPS group significantly upregulated the levels of MDA and iNOS in the tissues and significantly downregulated the levels of T-AOC, SOD, and GSH-Px (*p* < 0.05). In comparison with the LPS group, the PAMK + LPS group showed a significant increase in the SOD levels and a significant decrease in the MDA and iNOS levels in the tissues, also with *p* < 0.05 (Figure 3A–E).

### 3.5. Descriptive Analysis of Transcriptome Data in BF of Goslings

This experiment further explored the pathways involved in the role of PAMKs in alleviating LPS-induced BF damage in goslings. The BF tissues of goslings in the PAMK and PAMK + LPS groups were selected for the RNA-Seq transcriptome assay. As shown in Table 1, 46.6 to 54.6 million raw reads were generated for each sample. After the quality control filtering, each sample had 44.0 to 52.7 million valid reads, with a valid read ratio of over 94.36 for each sample. The base mass values of all samples ranged from 97.81% to 98.00%, the percentage of GC content was greater than 48.50%, and the sample data were tested by several indexes and shown to be qualified (Table 1).

### 3.6. Functional Enrichment Analysis of DEGs

**Figure 4:**
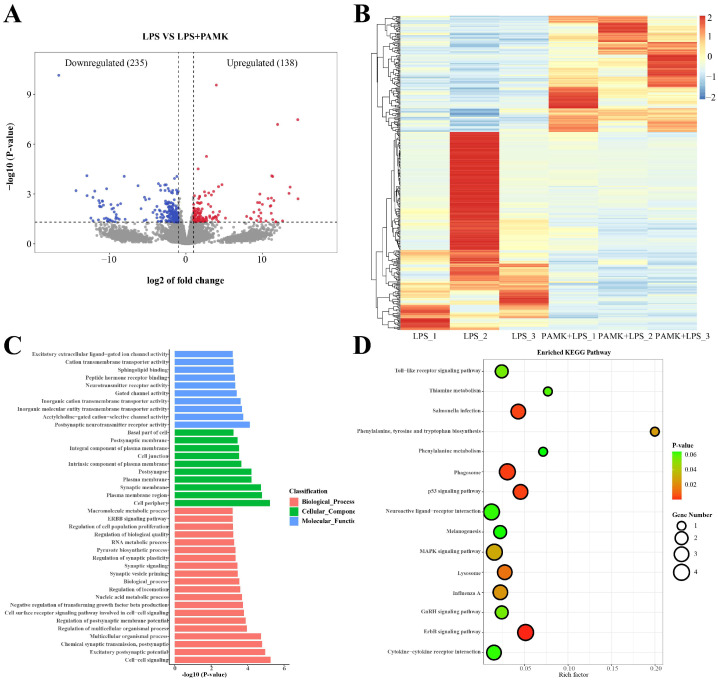
Map of DEGs in the BFs of the goslings from the LPS and PAMK + LPS groups. (**A**) PAMK + LPS vs. LPS DEGs volcano map. (**B**) Heat map. (**C**) GO histogram of the PAMK + LPS vs. LPS DEGs showing 40 significantly enriched GO terms. Horizontal coordinates indicate −log10 (*p*-value) and vertical coordinates indicate enriched GO terms. (**D**) KEGG bubble plot of the PAMK + LPS vs. LPS DEGs showing significantly enriched 15 pathways. Horizontal coordinates indicate *p*-value and vertical coordinates indicate enriched KEGG pathways.

A total of 373 DEGs were identified in this experiment, including 235 downregulated genes and 138 upregulated genes (Figure 4A,B).

To further determine the mechanism of the protective effect of PAMKs on the LPS-induced BF injury in the goslings, this experiment was performed to analyze the functional enrichment of the DEGs. The 40 GO terms enriched in the LPS group compared with the PAMK + LPS group included 20 GO terms for the BF, 10 GO terms for the Cellular Component (CC) analysis, and 10 GO terms for the Molecular Functional (MF) analysis. Among the 20 GO terms with a significantly enriched BF, the most enriched terms were related to intercellular signaling processes, such as the ErbB signaling pathway, the regulation of cell population proliferation, the regulation of synaptic plasticity, and synaptic signaling (*p* < 0.05) (Figure 4C). Among the 10 GO terms significantly enriched by the CC analysis, the DEGs were more involved in regions such as the basal part of the cell, postsynaptic membrane, and the integral components of the plasma membrane (*p* < 0.05). Among the 10 GO terms significantly enriched by the MF analysis, the DEGs were more enriched in excitatory extracellular ligand-gated ion channel activity, cation transmembrane transporter activity, sphingolipid binding, etc. (*p* < 0.05).

The results of the KEGG enrichment analysis show (Figure 4D) that these DEGs were more enriched in the KEGG pathways, mainly including the Toll-like receptor signaling pathway, salmonella infection, phenylalanine tyrosine and tryptophan biosynthesis, p53 signaling pathway, neuroactive ligand-receptor interactions, melanogenesis, MAPK signaling pathway, GnRH signaling pathway, ErbB signaling pathway, and cytokine–cytokine receptor interactions in these signaling pathways (*p* < 0.05).

### 3.7. PPI Network and qRT-PCR Method Validation Results

**Figure 5:**
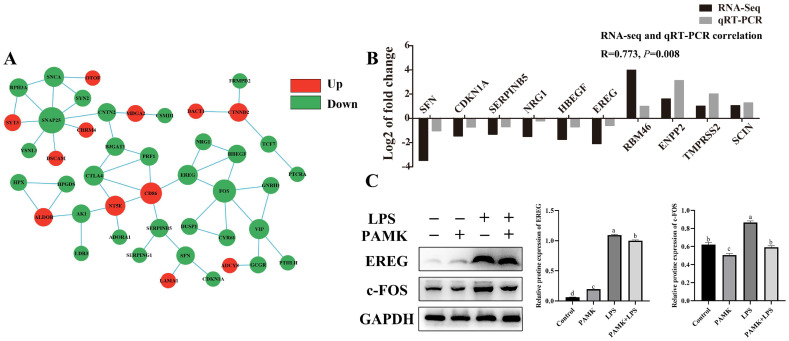
Identification and validation of the key DEGs. (**A**) Interaction map of the DEGs protein network. The sizes of the circles show the intensities of the data support, with red for PAMK + LPS vs. LPS upregulated genes and green for PAMK + LPS vs. LPS downregulated genes. (**B**) Results of qRT-PCR and RNA-Seq detection of two groups of DEGs. The log2 of the fold change is expressed as the mean value. (**C**) Verification of key genes in the EREG signaling pathway. Data are expressed as the mean ± standard error, and data columns labeled with different lowercase letters indicate significant differences (*p* < 0.05), and the same letter indicates that the differences are not statistically significant (*p* > 0.05).

The results of the PPI show that a total of 44 proteins were interlinked (Figure 5A). Among these 44 related proteins, there were 12 upregulated proteins and 32 downregulated proteins. To verify the accuracy of the RNA-Seq results, the relative expression levels of 10 DEGs (SFN, CDKN1A, SERPINB5, NRG1, HBEGF, EREG, RBM46, ENPP2, TMPRSS2, and SCIN) were randomly detected by a fluorescence quantification method in this experiment (Figure 5B). As shown in Figure 5, the expression changes of these DEGs demonstrated consistent upward or downward trends in both the RNA-Seq and qRT-PCR, confirming the reliability of the RNA-Seq data.

### 3.8. Key Gene Identification

Based on an in-depth analysis of the PPI network, we observed significant interactions of EREG with other proteins involved in stress response and damage repair. Preliminary bioinformatics analysis and functional prediction highlighted EREG’s important position and connections with key proteins, making it a candidate for further research. We hypothesized that PAMKs modulate the expression of EREG and its downstream signaling molecule c-FOS, and thus, plays a regulatory role in the cellular response to LPS induction. Therefore, this experiment employed WB to measure the relative expression and protein levels of EREG and the downstream gene c-FOS in the BF of goslings in the PAMK + LPS group compared with the LPS group (Figure 5C). The results show that compared with the LPS group, the PAMK + LPS group had significantly lower protein expression levels of EREG and c-FOS (*p* < 0.05). These findings suggest that PAMKs may exert a negative regulatory effect on the signaling pathways of these proteins by suppressing their expressions (Figure 5A).

### 3.9. The Impact of PAMKs on Cell Apoptosis and Cell Cycle in LPS Induced Injury to Gosling BF Cells

To further investigate the regulatory effect of the PAMKs on apoptosis in BF cells induced by LPS, an in vitro experiment was conducted. In this experiment, isolated BF cells were treated with LPS, and PAMKs were added for intervention. In this experiment, the isolated BF cells were treated with LPS administration at concentrations of 0.1, 1, 10, and 100 μg/mL. The results show that the addition of different concentrations of LPS significantly increased the expression of EREG compared with the control group (*p* < 0.05) (Figure S1A). Furthermore, the cell viability was lower than that of the control group at the LPS administration concentrations of 0.1 and 100 μg/mL (Figure S1B). The administration treatment of LPS resulted in an upregulation of the percentage of G0/G1 phase compared with the control group (Figure S1C,D). These results suggest that LPS can regulate the cell cycle and promote apoptosis by stimulating the activation of the key gene EREG. Next, this experiment chose to administer the treatment with LPS at a concentration of 0.1 μg/mL and add PAMKs for interference on top of this. The concentrations of the PAMKs were set to 1, 5, 10, 15, 20, 25, and 30 μg/mL. The results show that the addition of the PAMKs significantly alleviated the LPS-induced rise in the EREG expression (*p* < 0.05) (Figure S2A). The results of the cell viability assay show that the PAMKs had a positive effect on cell viability compared with the LPS group, with the optimal effect at PAMK administration concentrations of 10 and 20 μg/mL (Figure S2B). The results of the cell cycle show that the administration treatment of PAMKs downregulated the percentage of G0/G1 phase compared with the LPS group (Figure S2C,D).

**Figure 6:**
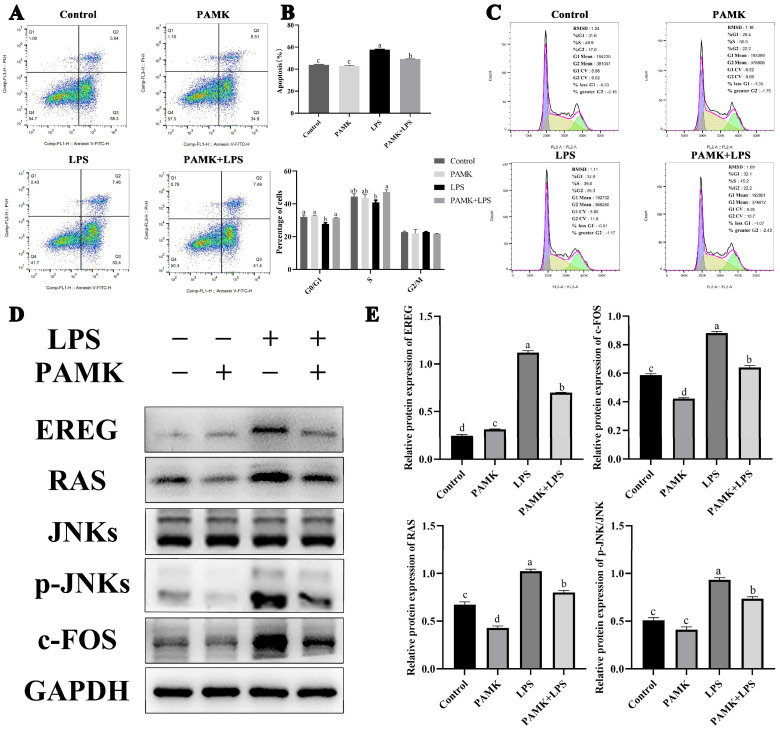
The impact of PAMKs on the cell apoptosis and cell cycle in LPS-induced injury to gosling BF cells. The effect of PAMKs on key genes of LPS-induced BF cell injury. (**A**) Cell apoptosis; (**B**) quantitative plots of the cell apoptosis and quantitative plots of the cell cycle; (**C**) cell cycle; (**D**) relative protein expressions of EREG, c-FOS, RAS, JNKs, and p-JNKs; (**E**) protein levels of EREG, RAS, c-FOS, JNKs, and p-JNKs. Data are expressed as the mean ± standard error, and data columns labeled with different lowercase letters indicate significant differences (*p* < 0.05), and the same letter indicates that the differences are not statistically significant (*p* > 0.05).

Therefore, subsequent studies chose to treat at a concentration of 0.1 μg/mL of LPS and added PAMKs for interference on top of that. The concentration of PAMKs was set to 10 μg/mL. The results show that compared with the control group, the LPS treatment significantly increased the occurrence of early and late apoptosis in the BF cells (*p* < 0.05). Furthermore, compared with the LPS group, the PAMKs significantly reduced the apoptosis rate of the BF cells. The cell cycle analysis revealed that in the LPS group, the number of cells in the G0/G1 phase and S phase was significantly reduced, while there was no significant difference in the G2/M phase. In contrast, in the PAMK + LPS group, the numbers of cells in the G0/G1 phase and S phase were significantly increased compared with the LPS group alone. These findings indicate that the addition of PAMKs to LPS-treated BF cells can ameliorate LPS-induced apoptosis and cell cycle disruption, demonstrating that PAMKs can regulate cellular processes to inhibit apoptosis in the bursa of Fabricius cells (Figure 6A–C).

### 3.10. Effect of PAMKs on EREG Signaling Pathway

The WB results show that the protein expression levels of EREG, c-FOS, p-JNKs, and RAS were significantly lower in the PAMK + LPS group than in the LPS group (*p* < 0.05), and the protein expression level of p-JNKs was also lower than that in the LPS group (Figure 6D,E). Notably, since there were two bands for JNKs and p-JNKs, the results of their densitometry analysis were the sum of the two bands.

## 4. Discussion

The BF in goslings is crucial for defense against pathogens and B lymphocyte function, and damage to the BF can lead to immune dysfunction and immune deficiencies [17,18]. When goslings ingest excess LPS, it may induce changes in the morphology and size of immune organs, disrupting their normal growth, development, and function, and severely impacting the poultry industry’s health and normal development. Research indicates that LPS induces tissue damage in the body primarily through two mechanisms: first, by affecting immune and epithelial cells and other target cells, causing abnormalities in surface molecules and increasing cell permeability, which leads to functional damage; and second, by activating immune cells to secrete a large amount of inflammatory mediators, thereby triggering inflammation [19]. Therefore, it is particularly important to protect the immune organs. The results revealed that the LPS caused a decrease in the volume and index of the BF organs in the goslings. This decrease was accompanied by a disorder in the arrangement of the BF follicles, a reduction in the density of lymphocytes in the cortex, and a decrease in the cortex-to-medulla ratio. These morphological and structural abnormalities disrupt the microenvironment necessary for the growth and development of B lymphocytes. The medullary area of the BF follicles is key for the development and differentiation of B lymphocytes, while the cortical area acts as a reservoir of mature B lymphocytes destined to migrate to peripheral immune tissues. Consequently, a reduction in the cortex-to-medulla ratio decreases the output of mature B lymphocytes, thereby reducing the immune function of the BF [20,21]. Crucially, the experimental results also show that the PAMKs significantly improved the decline in the BF organ index and morphological abnormalities caused by the LPS. By enhancing the immunity, the PAMKs may have helped restore the structure and function of the BF organs, thereby increasing the goslings’ ability to resist pathogens. This indicates that PAMKs can not only counteract the negative effects of LPS but also play a positive role in maintaining the health and stability of the goslings’ immune system.

In this study, there was a significant increase in the levels of pro-inflammatory cytokines IL-1β, IL-6, and TNF-α in the serum, along with a notable decrease in the levels of TGF-β. Additionally, the LPS induced a significant decrease in the levels of T-AOC, SOD, and GSH-Px and an increase in the levels of MDA and iNOS in the bronchus of the goslings. These outcomes suggest that LPS not only induces tissue injury in the BF but also triggered an activation of a gosling’s immune system. This activation led to the release of substantial amounts of inflammatory mediators, which, in turn, initiated an inflammatory response. The increase in inflammatory mediators further exacerbates oxidative stress, as these cytokines can induce more ROS production, forming a positive feedback loop that leads to further intensification of inflammation and oxidative damage [22]. LPS from bacteria binds to TLR4, triggering downstream inflammatory signaling pathways that produce inflammatory mediators like iNOS. These mediators subsequently activate a cascade of proinflammatory cytokines, including TNF-α, IL-1β, and IL-6 [23]. Studies showed that PAMKs activate T lymphocytes in the thymus through the novel_mir2/CTLA4/TCR and novel_mir2/CTLA4/CD28 signaling pathways, inhibiting the transcription of pro-inflammatory cytokines, such as IL-1β, IFN-γ, IL-4, and IL-10. It also enhances the expression of cytokines, like TGF-β, IL-6, and IL-5, helping to maintain the balance of cytokines in the body and alleviate immunosuppression [24,25,26]. In the LPS-stimulated BF, the PAMKs significantly reduced the levels of iNOS and MDA and decreased the production of these pro-inflammatory mediators. This reduction suggests that PAMKs may possess potent anti-inflammatory and antioxidant properties.

To further explore how PAMKs alleviate LPS-induced damage to the bursa of Fabricius in the goslings, this study utilized RNA-Seq technology to identify the DEGs in the BF from the goslings treated with LPS and PAMK + LPS. Functional enrichment analysis revealed that these DEGs were closely associated with intercellular signal transduction processes, particularly involving the ErbB signaling pathway, which plays a crucial role in immune responses and cellular communication. Further analysis using the KEGG pathway database indicated that the DEGs were significantly enriched in pathways such as the Toll-like receptor, p53, MAPK, GnRH, and ErbB signaling pathways. LPS activates the MAPK and ErbB signaling pathways by activating proteins in the EGF family, particularly EREG [27]. EREG was shown to exhibit greater bioactivity than other EGF family members and to bind to ErbB to initiate downstream signaling cascades [28,29,30]. The results of this study also observed that the expression of EREG protein in the BF of the LPS group was significantly increased, and the protein expression after intervention with PAMKs was significantly reduced compared with the LPS group. This indicates that PAMKs may alleviate the damage to the BF in goslings caused by LPS by suppressing the expression of EREG. The PPI network analysis revealed a strong biological interaction between EREG and FOS proteins, leading this study to identify the EREG signaling pathway within the MAPK signaling pathway as a key pathway, with EREG and c-FOS as potential target genes to explore the immunomodulatory mechanism of PAMKs.

**Figure 7:**
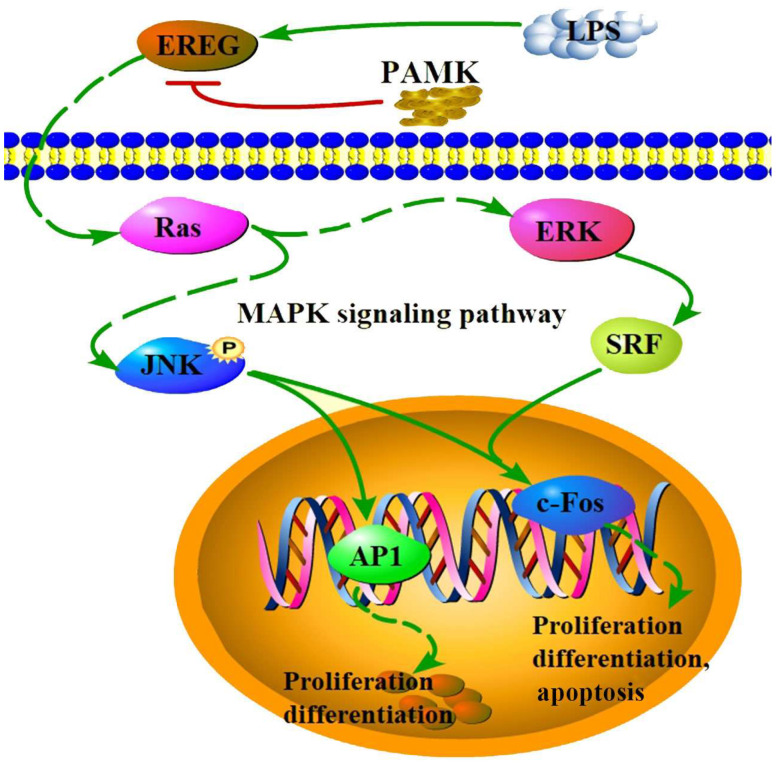
Diagram of the EREG and MAPK signaling pathways. Red arrows denote inhibition, green arrows denote promotion, solid lines represent direct interactions, and dashed lines represent indirect interactions.

To further verify the above results, the goslings’ BF cells were selected for isolation and culture. Through cell-cycle and apoptosis assays, the impact of the PAMKs on the LPS-induced alterations in the gosling BF cells was investigated. LPS can reduce the cell viability and cause cell-cycle arrest in a variety of cells, and it induces an inflammatory response and oxidative stress [31]. In the results of this experiment, the LPS dosing treatment caused a significant decrease in the cell viability of the BF cells by inducing a significant expression of EREG in the BF cells, which led to BF cell-cycle arrest, as well as promoting the phenomenon of apoptosis in the BF cells. The PAMKs regulated the expression levels of key proteins EREG and its downstream signaling molecules JNKs, p-JNKs, and c-FOS in the LPS-induced BF cells, which effectively alleviated the damage caused by the LPS. In the Western blot analysis, the observation of two bands for both JNKs and p-JNKs, indicative of different JNK isoforms, aligns with previous research findings [32,33]. It is noteworthy that the trend of these two bands is concordant, suggesting that these distinct JNK isoforms may be subject to similar regulatory mechanisms. The role of PAMKs significantly suppressed the protein expression levels of EREG in BF cells, which is crucial for enhancing cell viability, regulating cell cycle disorders, and inhibiting apoptosis within the cell cycle. EREG binds to the EGF receptor, activating the MAPK signaling pathway that includes p38 MAPK, JNKs, and Erk1/2, which can be stimulated by inflammation and stress [34]. Studies found that under the stimulation of pro-inflammatory cytokines, human granulosa cells may induce the biosynthesis of EREG, which further activates the MAPK signaling pathway [35]. Studies showed that EREG is not only involved in the interaction and positive feedback loops among growth factors but also amplifies inflammatory signals through the Ras-ERK pathway, especially playing a key role in the development and persistence of inflammatory diseases [36]. Furthermore, the JNK signaling pathway activated by EREG affects the ERK/p38 signaling pathway, which may be associated with the progression of gastric cancer in certain cases [37]. The JNK family comprises multiple subtypes, JNK1, JNK2, and JNK3, all of which play significant roles in cellular apoptosis and immune-inflammatory responses. Research indicates that JNK1 is primarily involved in responding to oxidative stress and promoting apoptosis, JNK2 plays a crucial role in the activation of immune cells and inflammatory reactions, and JNK3 is instrumental in the apoptosis of neuronal cells [38]. JNK induces the phosphorylation of c-Jun and an increase in c-Fos protein levels, thereby activating the AP-1 transcription factor. Transgenic compounds induce cell death and mitotic arrest in triple-negative breast cancer cells through the activation of AP-1 in vitro [39]. This regulation significantly impacts cell proliferation and apoptosis, with the JNKs signaling pathway playing a particularly critical role in the inflammatory response. It controls the immune response and inflammatory processes by affecting the expression of inflammatory cytokines and chemokines [40,41]. In this study, PAMKs promoted JNKs phosphorylation, affecting the expression of c-Fos and the activity of AP-1, which effectively regulated the LPS-induced damage in the gosling bursa cells (Figure 7).

## 5. Conclusions

PAMKs may promote immune regulation, anti-inflammation, and antioxidant stress to alleviate LPS-induced BF injury by inhibiting EREG expression.

## Figures and Tables

**Table 1:** Quality analysis of transcriptome sequencing and mapping.

Sample	Raw Data (Reads)	Valid Data (Reads)	Valid Ratio (Reads)	Q30%	GC Content%
LPS-1	54,625,942	52,785,740	96.63	97.93	47.50
LPS-2	4,652,940	44,020,058	94.36	97.81	50.00
LPS-3	51,574,198	49,798,384	96.56	98.00	48.50
PAMK + LPS-1	53,332,552	51,434,548	96.44	97.98	49.50
PAMK + LPS-2	50,618,118	48,819,648	96.45	97.97	48.50
PAMK + LPS-3	48,542,906	46,660,038	96.12	97.97	48.50

## Data Availability

The data presented in this study are openly available in the Sequence Read SRA: PRJNA1004021.

## Supplementary Materials

The following supporting information can be downloaded at: www.mdpi.com/10.3390/ani15010084/s1, Figure S1: Effects of different concentrations of LPS on BF cells. Figure S2: Alleviation of LPS-induced BF cells injury by different concentrations of PAMKs. Table S1. Primer information.
