# Supplementary material for: Polysaccharides of Atractylodes Macrocephala Koidz Alleviate LPS-Induced Bursa of Fabricius Injury in Goslings by Inhibiting EREG Expression
Source: Animals (Basel). 2025 Jan 2;15(1):84. doi: 10.3390/ani15010084 (PMC11718795; doi:10.3390/ani15010084)
Supplement: Supplementary file 1 [file animals-15-00084-s001.zip › animals-3330274-supplementary.pdf]

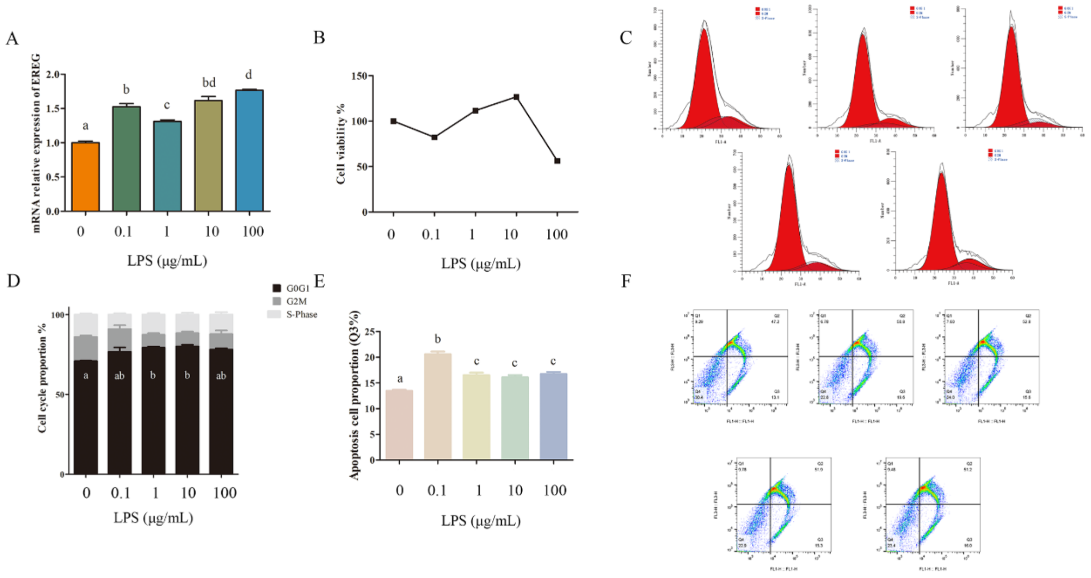

Figure S1 Effect of different concentrations of LPS on BF cells (A) Effect of different concentrations of LPS on EREG gene expression in BF cells. (B) Cells viability. (C) Cells cycle. (D) Quantitative plots of cells cycle. (E) Quantitative plots of cells apoptosis. (F) Cells apoptosis.

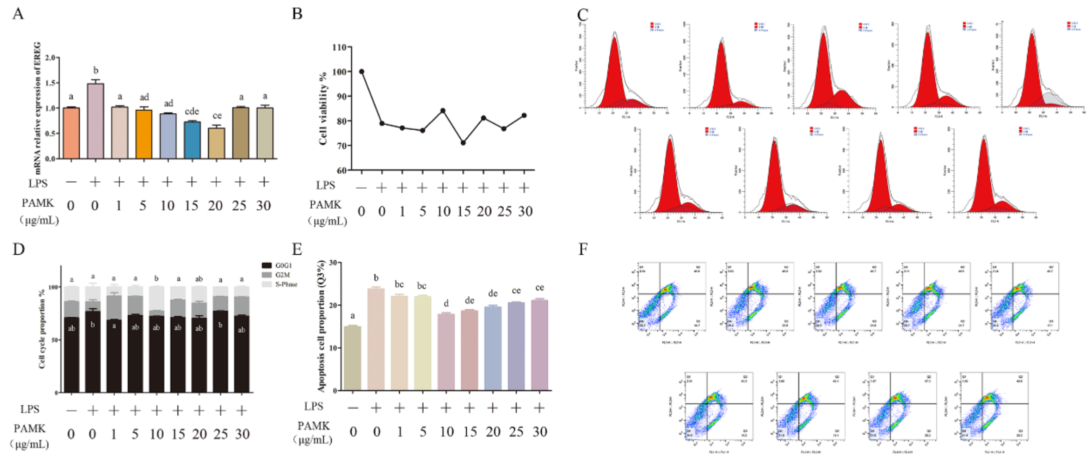

Figure S2 Alleviation of LPS-induced BF cells injury by different concentrations of PAMK(A) Effect of different concentrations of PAMK on 0.1 μg/mL LPS-induced EREG gene expression in BF cells. (B) Cells viability. (C) Cells cycle. (D) Quantitative plots of cells cycle. (E) Quantitative plots of cells apoptosis. (F) Cells apoptosis.
