# Supplementary material for: Polysaccharides of Atractylodes Macrocephala Koidz Alleviate LPS-Induced Bursa of Fabricius Injury in Goslings by Inhibiting EREG Expression
Source: Animals (Basel). 2025 Jan 2;15(1):84. doi: 10.3390/ani15010084 (PMC11718795; doi:10.3390/ani15010084)
Supplement: Supplementary file 1 [file animals-15-00084-s001.zip › Table S1 Primer information (proof).docx]

Table S1 Primer information

| Genes | Accession number | Primer sequences(5’→3’) |
| --- | --- | --- |
| *EREG* | XM_066996592.1 | F: CAGGTGTGATGTCGGTTTCT |
|  |  | R: GCACCGACCTCCTTGTATTC |
| *SFN* | XM_013199620.3 | F: GGCTTGCTGGACAAGTATCT |
|  |  | R: TCGTAGTGGAAGACGGA-GAA |
| *CDKN1A* | XM_048054538.2 | F: GGGCTCCAGAATGACTTTGA |
|  |  | R: GTCCTTAGATGGGACCTT-GTG |
| *SERPINB5* | XM_013187398.3 | F: CCAGAAGCAGAGATCAAAGAA |
|  |  | R: CTGGGATTGGTCCACTG-TAATAG |
| *HBEGF* | XM_066977025.1 | F: ATGGAGAAGACTGGAGAGAGA |
|  |  | R: CCCTCCTGTGCCTAAGAAAG |
| *RBM46* | XM_013175184.3 | F: CTGGGAAGATCTACGAGTTTAG |
|  |  | R: CTGCTCTGTGG-GACTCATATTC |
| *ENPP2* | XM_048071290.2 | F: TGCTGCCAGGATCTTTAGG |
|  |  | R: CCGTTATTAGGAGCAGGCTTTA |
| *SCIN* | XM_048075408.2 | F: AGAGTCAGCCAAGGCAAAG |
|  |  | R: CATCCAGGTG-TAGCCAGAATTA |
| *TMPRSS2* | XM_048071483.2 | F: CCCATATCCACCGTACTTCTC |
|  |  | R: CCCGAAGACCCACACTC-TATTA |
| *NRG1* | XM_066987722.1 | F: CGTTCCGTCTCTGGAATCTG |
|  |  | R: CATTGCTCCTCTCGTTGTCT |
| *ACTB* | XM_066977989.1 | F: GCACCCAGCACGATGAAAAT |
|  |  | R: GACAATGGAGGGTCCGGATT |
